# Supplementary material for: A fault-intrusion-tolerant system and deadline-aware algorithm for scheduling scientific workflow in the cloud
Source: PeerJ Comput Sci. 2021 Nov 2;7:e747. doi: 10.7717/peerj-cs.747 (PMC8576554; doi:10.7717/peerj-cs.747)
Supplement: Supplemental Information 6 [file peerj-cs-07-747-s006.zip › Myproject1.1/docs/org/cloudbus/cloudsim/class-use/ResCloudlet.html]

Uses of Class org.cloudbus.cloudsim.ResCloudlet


JavaScript is disabled on your browser.


- Overview
- Package
- Class
- Use
- Tree
- Deprecated
- Index
- Help

- Prev
- Next

- Frames
- No Frames

- All Classes

## Uses of Class org.cloudbus.cloudsim.ResCloudlet

- Packages that use ResCloudlet

  | Package | Description |
  | --- | --- |
  |  |  |
  | --- | --- |
  | org.cloudbus.cloudsim |  |
  | org.cloudbus.cloudsim.lists |  |
  | org.cloudbus.cloudsim.network.datacenter |  |
- - ### Uses of ResCloudlet in org.cloudbus.cloudsim

    Methods in org.cloudbus.cloudsim with type parameters of type ResCloudlet

    | Modifier and Type | Method and Description |
    | --- | --- |
    |  |  |
    | --- | --- |
    | `protected <T extends ResCloudlet>  void` | CloudletSchedulerSpaceShared.`cloudletWaitingList(java.util.List<T> cloudletWaitingList)` Cloudlet waiting list. |
    | `protected <T extends ResCloudlet>  java.util.List<T>` | CloudletSchedulerSpaceShared.`getCloudletExecList()` Gets the cloudlet exec list. |
    | `protected <T extends ResCloudlet>  java.util.List<T>` | CloudletSchedulerTimeShared.`getCloudletExecList()` Gets the cloudlet exec list. |
    | `protected <T extends ResCloudlet>  java.util.List<T>` | CloudletSchedulerSpaceShared.`getCloudletFinishedList()` Gets the cloudlet finished list. |
    | `protected <T extends ResCloudlet>  java.util.List<T>` | CloudletSchedulerTimeShared.`getCloudletFinishedList()` Gets the cloudlet finished list. |
    | `protected <T extends ResCloudlet>  java.util.List<T>` | CloudletSchedulerSpaceShared.`getCloudletPausedList()` Gets the cloudlet paused list. |
    | `protected <T extends ResCloudlet>  java.util.List<T>` | CloudletSchedulerTimeShared.`getCloudletPausedList()` Gets the cloudlet paused list. |
    | `protected <T extends ResCloudlet>  java.util.List<T>` | CloudletSchedulerSpaceShared.`getCloudletWaitingList()` Gets the cloudlet waiting list. |
    | `protected <T extends ResCloudlet>  void` | CloudletSchedulerSpaceShared.`setCloudletExecList(java.util.List<T> cloudletExecList)` Sets the cloudlet exec list. |
    | `protected <T extends ResCloudlet>  void` | CloudletSchedulerTimeShared.`setCloudletExecList(java.util.List<T> cloudletExecList)` Sets the cloudlet exec list. |
    | `protected <T extends ResCloudlet>  void` | CloudletSchedulerSpaceShared.`setCloudletFinishedList(java.util.List<T> cloudletFinishedList)` Sets the cloudlet finished list. |
    | `protected <T extends ResCloudlet>  void` | CloudletSchedulerTimeShared.`setCloudletFinishedList(java.util.List<T> cloudletFinishedList)` Sets the cloudlet finished list. |
    | `protected <T extends ResCloudlet>  void` | CloudletSchedulerSpaceShared.`setCloudletPausedList(java.util.List<T> cloudletPausedList)` Sets the cloudlet paused list. |
    | `protected <T extends ResCloudlet>  void` | CloudletSchedulerTimeShared.`setCloudletPausedList(java.util.List<T> cloudletPausedList)` Sets the cloudlet paused list. |

    Methods in org.cloudbus.cloudsim with parameters of type ResCloudlet

    | Modifier and Type | Method and Description |
    | --- | --- |
    |  |  |
    | --- | --- |
    | `abstract void` | CloudletScheduler.`cloudletFinish(ResCloudlet rcl)` Processes a finished cloudlet. |
    | `void` | CloudletSchedulerDynamicWorkload.`cloudletFinish(ResCloudlet rcl)` Processes a finished cloudlet. |
    | `void` | CloudletSchedulerSpaceShared.`cloudletFinish(ResCloudlet rcl)` Processes a finished cloudlet. |
    | `void` | CloudletSchedulerTimeShared.`cloudletFinish(ResCloudlet rcl)` Processes a finished cloudlet. |
    | `double` | CloudletSchedulerDynamicWorkload.`getEstimatedFinishTime(ResCloudlet rcl, double time)` Get estimated cloudlet completion time. |
    | `abstract double` | CloudletScheduler.`getTotalCurrentAllocatedMipsForCloudlet(ResCloudlet rcl, double time)` Gets the total current allocated mips for cloudlet. |
    | `double` | CloudletSchedulerDynamicWorkload.`getTotalCurrentAllocatedMipsForCloudlet(ResCloudlet rcl, double time)` Gets the current mips. |
    | `double` | CloudletSchedulerSpaceShared.`getTotalCurrentAllocatedMipsForCloudlet(ResCloudlet rcl, double time)` |
    | `double` | CloudletSchedulerTimeShared.`getTotalCurrentAllocatedMipsForCloudlet(ResCloudlet rcl, double time)` |
    | `abstract double` | CloudletScheduler.`getTotalCurrentAvailableMipsForCloudlet(ResCloudlet rcl, java.util.List<java.lang.Double> mipsShare)` Gets the total current mips for the Cloudlet. |
    | `double` | CloudletSchedulerDynamicWorkload.`getTotalCurrentAvailableMipsForCloudlet(ResCloudlet rcl, java.util.List<java.lang.Double> mipsShare)` Gets the total current mips for the clouddlet. |
    | `double` | CloudletSchedulerSpaceShared.`getTotalCurrentAvailableMipsForCloudlet(ResCloudlet rcl, java.util.List<java.lang.Double> mipsShare)` |
    | `double` | CloudletSchedulerTimeShared.`getTotalCurrentAvailableMipsForCloudlet(ResCloudlet rcl, java.util.List<java.lang.Double> mipsShare)` |
    | `abstract double` | CloudletScheduler.`getTotalCurrentRequestedMipsForCloudlet(ResCloudlet rcl, double time)` Gets the total current requested mips for cloudlet. |
    | `double` | CloudletSchedulerDynamicWorkload.`getTotalCurrentRequestedMipsForCloudlet(ResCloudlet rcl, double time)` Gets the current mips. |
    | `double` | CloudletSchedulerSpaceShared.`getTotalCurrentRequestedMipsForCloudlet(ResCloudlet rcl, double time)` |
    | `double` | CloudletSchedulerTimeShared.`getTotalCurrentRequestedMipsForCloudlet(ResCloudlet rcl, double time)` |
    | `void` | CloudletSchedulerDynamicWorkload.`updateUnderAllocatedMipsForCloudlet(ResCloudlet rcl, double mips)` Update under allocated mips for cloudlet. |
  - ### Uses of ResCloudlet in org.cloudbus.cloudsim.lists

    Methods in org.cloudbus.cloudsim.lists with type parameters of type ResCloudlet

    | Modifier and Type | Method and Description |
    | --- | --- |
    |  |  |
    | --- | --- |
    | `static <T extends ResCloudlet>  ResCloudlet` | ResCloudletList.`getByIdAndUserId(java.util.List<T> list, int cloudletId, int userId)` Returns a given Cloudlet. |
    | `static <T extends ResCloudlet>  int` | ResCloudletList.`getPositionById(java.util.List<T> cloudletList, int id)` Returns the position of the cloudlet with that id, if it exists. |
    | `static <T extends ResCloudlet>  int` | ResCloudletList.`indexOf(java.util.List<T> list, int cloudletId, int userId)` Finds the index of a Cloudlet inside the list. |
    | `static <T extends ResCloudlet>  boolean` | ResCloudletList.`move(java.util.List<T> listFrom, java.util.List<T> listTo, T cloudlet)` Move a ResCloudlet object from this linked-list into a specified one. |

    Methods in org.cloudbus.cloudsim.lists that return ResCloudlet

    | Modifier and Type | Method and Description |
    | --- | --- |
    |  |  |
    | --- | --- |
    | `static <T extends ResCloudlet>  ResCloudlet` | ResCloudletList.`getByIdAndUserId(java.util.List<T> list, int cloudletId, int userId)` Returns a given Cloudlet. |
  - ### Uses of ResCloudlet in org.cloudbus.cloudsim.network.datacenter

    Methods in org.cloudbus.cloudsim.network.datacenter with type parameters of type ResCloudlet

    | Modifier and Type | Method and Description |
    | --- | --- |
    |  |  |
    | --- | --- |
    | `protected <T extends ResCloudlet>  void` | NetworkCloudletSpaceSharedScheduler.`cloudletWaitingList(java.util.List<T> cloudletWaitingList)` Cloudlet waiting list. |
    | `protected <T extends ResCloudlet>  java.util.List<T>` | NetworkCloudletSpaceSharedScheduler.`getCloudletExecList()` Gets the cloudlet exec list. |
    | `protected <T extends ResCloudlet>  java.util.List<T>` | NetworkCloudletSpaceSharedScheduler.`getCloudletFinishedList()` Gets the cloudlet finished list. |
    | `protected <T extends ResCloudlet>  java.util.List<T>` | NetworkCloudletSpaceSharedScheduler.`getCloudletPausedList()` Gets the cloudlet paused list. |
    | `protected <T extends ResCloudlet>  java.util.List<T>` | NetworkCloudletSpaceSharedScheduler.`getCloudletWaitingList()` Gets the cloudlet waiting list. |
    | `protected <T extends ResCloudlet>  void` | NetworkCloudletSpaceSharedScheduler.`setCloudletExecList(java.util.List<T> cloudletExecList)` Sets the cloudlet exec list. |
    | `protected <T extends ResCloudlet>  void` | NetworkCloudletSpaceSharedScheduler.`setCloudletFinishedList(java.util.List<T> cloudletFinishedList)` Sets the cloudlet finished list. |
    | `protected <T extends ResCloudlet>  void` | NetworkCloudletSpaceSharedScheduler.`setCloudletPausedList(java.util.List<T> cloudletPausedList)` Sets the cloudlet paused list. |

    Methods in org.cloudbus.cloudsim.network.datacenter with parameters of type ResCloudlet

    | Modifier and Type | Method and Description |
    | --- | --- |
    |  |  |
    | --- | --- |
    | `void` | NetworkCloudletSpaceSharedScheduler.`cloudletFinish(ResCloudlet rcl)` Processes a finished cloudlet. |
    | `double` | NetworkCloudletSpaceSharedScheduler.`getTotalCurrentAllocatedMipsForCloudlet(ResCloudlet rcl, double time)` |
    | `double` | NetworkCloudletSpaceSharedScheduler.`getTotalCurrentAvailableMipsForCloudlet(ResCloudlet rcl, java.util.List<java.lang.Double> mipsShare)` |
    | `double` | NetworkCloudletSpaceSharedScheduler.`getTotalCurrentRequestedMipsForCloudlet(ResCloudlet rcl, double time)` |

- Overview
- Package
- Class
- Use
- Tree
- Deprecated
- Index
- Help

- Prev
- Next

- Frames
- No Frames

- All Classes
